# Making space for the new state capitalism, part II: Relationality, spatiotemporality and uneven development

**DOI:** 10.1177/0308518X231156913

**Published:** 2023-02-12

**Authors:** Ilias Alami, Heather Whiteside, Adam D Dixon, Jamie Peck

**Affiliations:** Department of Human Geography, 8097 Uppsala University, Uppsala, Sweden; Department of Political Science, 8430 University of Waterloo, Waterloo, Canada; Faculty of Arts and Social Sciences, 5211 Maastricht University, Maastricht, the Netherlands; Department of Geography, University of British Columbia, Vancouver, Canada

**Keywords:** State capitalism, uneven development, variegated capitalism, territory, spatiotemporality

## Abstract

The theme issue ‘Making Space for the New State Capitalism’ brings together insights from critical economic geography and heterodox political economy through a series of papers published in three installments, each accompanied by an introductory essay written by the guest editors. In this, the second of these introductory commentaries, we explore the consequences of embracing relationality, spatiotemporality and uneven development, together with the second group of papers. Introducing a final group of papers, the third installment will address the challenges and opportunities of thinking conjuncturally.

## Introduction

This introductory essay to the second installment of the theme issue ‘Making Space for the New State Capitalism’ continues our theory-driven reflections on the state of state capitalism studies. We focus in particular on the analytical reorientation that the field has recently experienced, with a growing body of literature (in this theme issue and elsewhere) emphasizing the role and significance of relationality, spatial (inter)connections and geographical and temporal unevenness in the making of contemporary state capitalism. In different ways, the contributions offered here reflect the theoretical proclivities, methodological inclinations and thematic preferences of geographical political economists sensitive to relational spatiotemporality and uneven development. Conceived as a flexible set of critical interrogations concerning the changing role of the state and the re-articulation of state-capital relations at the current historical juncture, rather than a fully formed concept or model, the *problematic* of ‘new’ state capitalism is taken up by geographical political economists in ways that enrich the field and push it in new and exciting directions (see Alami et al., 2022a; Whiteside et al., 2023).

One of the original motivations for this theme issue, in fact, was to address the need to ‘spatialize’ state capitalism, indeed to capitalize upon this opportunity. Moreover, the spatialization of state capitalism is equally a reflection of the momentous political-economic shifts that have come to increasingly shape research priorities as of late. From pandemics and financial crashes to supply chain crunches, food insecurity, energy crisis, rampant inflation, outright wars and escalating climate change, there have in recent years been many consequential, and at times bewildering, state transformations across geographic space and policy realms, quite often defying received categorizations of capitalist institutional diversity. Generic images of neoliberal capitalism and state capitalism, constructed as incompatible models of development based on fundamentally different logics, rationalities and principles, appear strikingly inadequate to represent and explain the multifaceted processes of state restructuring unfolding before our eyes. For instance, the hypothesis that state capitalism is a phenomenon neatly contained within the geographic space of emerging market economies (and outside the core of the world capitalist economy) (cf. Kurlantzick, 2016; Ricz, 2021), together with the proposition that the state in (neo)liberal economies plays a largely market-facilitating role (cf. Chapter 3 in Harvey, 2005; Plant, 2010), are now much less convincing than they may once have been. In short, assumptions about the geographic coordinates of state capitalism have been buckling under the weight of contemporary events, the terraforming dynamics of which plainly exceed the purchase of static and essentialized models (or varieties) of capitalism.

Recent developments have made clear that contemporary state capitalism is a deeply geographical affair, characterized by relational transformations across space and variegated socio-spatial and territorial outcomes (Alami, 2022). The transnational impacts of ‘quantitative tightening’ in the United States, China's green industrial policy and the war in Ukraine show that a particular form of state activism in a specific site or jurisdiction may have cascading impacts and ricocheting effects across territorial borders, including in non-adjacent locations and faraway places, insofar as they spark a policy response on the part of states which are directly affected. Effects may be less direct (but no less significant) when a particular modality of state intervention in one place transforms economic conditions and sociopolitical relations elsewhere, in turn prompting other states to react to this new context by developing novel prerogatives, capacities, institutional forms and modes of economic practice. The picture that emerges, then, is hardly one of a mosaic of neatly demarcated varieties of capitalism. Neither does it resemble a zero-sum contest between one model and another. Instead, moving landscapes of state intervention are being created, constituted, transformed and recombined over time through interconnected socio-spatial processes and interactions. Questions of space and geography are clearly fundamental to understanding ongoing patterns of state restructuring, and if the field of new state capitalism studies is to continue delivering salient theoretical insights, research hypotheses and empirical observations, it must come to grips with the geographical limitations of the first-generation models (and imaginaries) that initially animated the field. We suggest that this will require the development of more relational-processual approaches, emphasizing non-linear movements and trajectories, as opposed to the analysis of fixed or self-contained systems (Alami and Dixon, 2023; Peck, 2021).

The six articles featured in this second installment of the theme issue all advance the plenary claim that geography matters to the study of present-day state capitalism. In ‘State Capitalism and Spanish port development along the Maritime Silk Road’, Jensen (2023) studies the dynamic interactions between Spanish and Chinese state-capitalist trajectories and their material expression in maritime transport infrastructure, based on a case study of the port of Valencia in Spain and Chinese state-owned COSCO shipping group. While Chinese state-owned shipping enterprises and Spanish port authorities have varied political-economic strategies, these have not been mutually exclusive or incompatible. Synergies between the two increasingly shape port development and macro-regional logistical networks in Europe, in the context of acute competitive pressures from global shipping markets (Jensen, 2023).

Kinossian and Morgan's (2023) analysis of two state-sponsored megaprojects in Russia – the upgrade of the Northern Sea Route and the construction the of Skolkovo Innovation Centre – uncovers their role in the reproduction of an authoritarian form of state capitalism. State-sponsored megaprojects serve not only the objectives of territorial control but also spatial development strategies. As such, they embody the state's attempts to modernize its economic base in order to mobilize the resources necessary to maintain economic and political stability. They consequently act as the linchpin of a multi-scalar reconfiguration of state power and facilitate the consolidation of authoritarian tendencies.

In ‘A very British state capitalism’, Wood et al. (2023) argue that the uneven distribution of COVID-19 financial assistance across regions and sectors has exacerbated spatial variegation and unequal territorial outcomes in the British variant of state capitalism. Their study of the granting of government bailouts and contracts emphasizes the significance of both sectoral concerns and political connections, and shows that the British case offers a peculiar combination of features traditionally associated with different varieties of capitalism (crony, liberal and state capitalism). By underlining the interpenetration of these logics/models in space and their hybridization, Wood et al.'s (2023) analysis problematizes rigid dichotomizations of discrete cases and categories.

Su and Lim (2023) offer an examination of how and why Yunnan, a south-western Chinese province that was economically marginalized for several decades, emerged as a geostrategic platform for facilitating new rounds of capital accumulation. The study of Chinese state spatial strategies to revalorize Yunnan suggests that territoriality – or the use of territory for political, economic and social ends – plays a key role in reconfiguring what Su and Lim call the ‘sovereignty–accumulation nexus.’ This case exemplifies that emerging state-capitalist configurations must be seen as new institutional articulations of capital accumulation, territoriality and the reproduction of state sovereignty.

The notion of territoriality is also mobilized in ‘Hybrid governance and extraterritoriality’, where McGregor and Coe (2023) explore the implications of the new state capitalism for global production networks. Focusing on the case of Singapore's state-owned entities in oil global production networks, they show that the internationalization of state-capital hybrids such as state-owned firms and sovereign funds adds an extraterritorial dimension to the state. Indeed, the system of ‘hybrid public–private governance’ that results (in both corporate and network terms) allow the state to influence domestic outcomes as well as global production network configurations beyond its borders.

Ward et al. (2023) offer a study of sovereign wealth fund investment into London real estate, which they position in the context of city governance, the geopolitics of real estate and resultant relational forms of regulation. Focusing on Qatari Investment Authority's involvement in London's Olympic Village, they demonstrate that configurations of state capitalism in this case result from the strategic coupling of intersecting state spatial projects, notably those of the Qatari state and of UK governance actors.

Although highly diverse in terms of theoretical claims, analytical themes, geographical terrains and empirical concerns, what these articles all show is that spatializing the new state capitalism consists neither of simply ‘adding’ space to state capitalism studies, nor does it mean invoking space in the form of the passive background for the front-of-stage maneuvres of state-capitalist actors. Rather, to paraphrase Sheppard (2011), spatiality is endogenized into state capitalism, meaning that it is conceived in terms of the constitutive properties of contemporary state capitalism. Thinking through spatiality, from this perspective involves investigating at least three moments: (1) state capitalism as space-dependent, meaning that it is conditioned by geographical unevenness (e.g. political-territorial configurations together with the spatial distribution of economic phenomena such as divisions of labour, the location of key resources, assets, firms and markets, the accumulation of wealth and power, the concentration of command functions and the flow of commodities, finance and information); (2) the reproduction of state capitalism through socio-spatial processes and relations; and (3) the production of distinctively state-capitalist geographies, including scalar configurations, territories, built environments and geographical landscapes (infrastructure, urbanization, megaprojects, etc.), which in turn condition the uneven geographical development of state capitalism. In short, the new state capitalism can only be understood in the context of ongoing processes of *uneven development*.

In the remainder of this essay, we offer a series of geographical provocations, with the hope that these will help consolidate the recent analytical reorientation of new state capitalism studies narrated earlier. In particular, we submit that the core intellectual project of state capitalism studies would greatly benefit from a more systematic exploration of the foundational role of ‘space-as-process’ (Swyngedouw, 2000: 45) in the making and unfolding of present-day state capitalism. We suggest that three tools of critical socio-spatial analysis can be especially useful in this respect: (1) relationality, (2) scale and (3) territory. Socio-spatial analysis can help researchers develop theoretical reconstructions and empirical investigations which engage and problematize the role of space and time in the uneven development of contemporary state capitalism. Understood in the context of uneven development, state capitalism as *problematic* tackles a sophisticated range of processes, from spatial differentiation and geo-institutional variegation to divergent rates of capital accumulation and spasmodic state transformations across the spaces of the capitalist world economy.

That said, even the most productive intellectual developments in a field of inquiry often produce new tensions and limitations, and the spatialization of state capitalism is no exception. We therefore argue that the future of state capitalism studies will be shaped by our collective ability to develop new research strategies, designs and methodologies in order to confront these challenges in both empirical and theoretical terms.

## Centring relationality in the new state capitalism

Relational thinking is forefront in the various intellectual traditions of economic geography (cf. Harvey, 1982; Massey, 1984), and the discipline has also experienced a series of relational turns (Jones, 2009; Murphy, 2018; Yeung, 2005). In fact, ‘it is hard not to think of geographical problems in relational terms – whether implicitly or explicitly’ (Yeung, 2005: 38). Relationality does not simply denote a concern with the ways in which two or more things, actors, or processes are connected and enmeshed in a web of relations. It is about seeing social entities and processes as the product of a set of interrelationships. Relations, then, are not a sort of passive condition, or background context in which social entities exist or events merely take place, but an active force that enters into the very constitution of political-economic actors, organizational forms and restructuring processes. Social entities are intrinsically constituted by relations of connection and interaction with others. They have no existence prior to (and outside of) these relations and the wider environment in which they are embedded, nor do they possess an ‘independent’ existence, such that they can be defined and accounted for solely with reference to ‘internal’ characteristics. As such, they exist and express themselves as relational entities. Their evolution through time and space is likewise shaped by shifting constellations of socio-spatial relations. Indeed, social entities develop and transform themselves through the working and reworking of these relationships over time. In sum, relationality entails *relational-processual* modes of thinking.

A telling illustration of this mode of thinking can be found in the conception of capitalism advanced by Anievas and Nisancioglu:

To say what capitalism ‘is’ runs the risk of reducing capitalism to a thing, which tends to obscure the multivalent connections in society that facilitate, structure and ultimately limit its reproduction. More specifically, it carries the implication that any given social factor contains an essence that is logically independent of other factors to which it is related. … [Capitalism is shaped by] historically specific configurations of social relations and processes. Such a relational-processual [conception] helps us move away from ‘abstract one-sided’ self-representations of capitalism [so as to see] capitalism not as a fixed entity, but as one that morphs and reconfigures social relations according to certain historical problems, challenges, struggles, contradictions, limits and opportunities (Anievas and Nisancioglu, 2015: 8–9).

In the company of Anievas and Nisancioglu, we see a role here for ‘configurative’ conceptions of capitalism, as opposed to more reductionist, singular, tranhistorical and ‘universal’ approaches. If capitalism is understood, in such terms, as a *necessarily* variegated phenomenon, there is a corresponding imperative to map and theorize across historical and geographical configurations (see Peck, 2023). While the criteria for such alternative theorizations must surely remain open, plural and contestable, there are reasons to be particularly skeptical of approaches premised (explicitly or implicity) on a singular conception of ‘advanced’ capitalism, or that sort capitalisms according to a spectrum running from ‘free market’ to ‘statist’ configurations. Similarly, there are reasons to be skeptical of conceptions of capitalist varieties as relatively coherent, bounded units governed by their own intrinsic logics.

For state capitalism research, this means at least two things. First, the landscapes of state capitalism must not be seen as a collection of self-contained or self-standing entities (whether varieties of capitalism, repertoires of state action, class-political projects and so on) with internal coherence outside of their interconnections and interrelationships. Rather, they must be conceived as constructed and emerging through those relations, and linked together in their mutual constitution. Put differently, they must not be seen as relatively autonomous ‘building blocs’ of state capitalism, but as co-created and part and parcel of a complex, relational whole. Second, to embrace relationality in the study of state capitalism is to demand modes of analysis that uncover *the ways in which relational co-constitution shapes the form, logics and outcome of emerging state-capitalist landscapes*.

This means examining emergent repertoires of state intervention in terms of dynamic and heterogenous interconnected socio-spatial processes and interactions linking state-capitalist actors to particular institutional contexts and their embeddedness in wider socio-spatial structures and patterns of uneven development. The mutual shaping of trajectories of techno-industrial and innovation policy in the USA, China, Germany and other western European countries are notable instances of competitive relations resulting in an extension of state prerogatives and policy interventions (e.g. see Baltz, 2022; Germann, 2022; Rolf and Schindler, 2023). Relations between state transformations can also be more synergistic, as demonstrated in Jensen's work (2023). Other examples include the mutually beneficial relation developing between governments in Central and Eastern Europe and Chinese state-owned enterprises, which have influenced patterns of state formation (notably the consolidation of illiberal political-economic regimes) and the development of large-scale infrastructure projects (Rogers, 2022; Szabó and Jelinek, 2023).

Tracing the interrelation, co-evolution and interaction between repertoires of state intervention thus offers insight into political multiplicity as a driver of present-day state capitalism. Relationality is also a means to deconstruct some of the static and mutually exclusive geographical imaginaries that have informed previous writings on supposedly discrete national state capitalisms, and drawing connections between cases invites more fine-grained understandings of state-capitalist politics as connections that breed both conflict and collaboration. By virtue of its insistence on connection and interdependence, relationality prompts comparison and problematizes positionality, helping to negotiate the tension in state capitalism studies discussed in our previous introductory essay (see Whiteside et al., 2023) between site-specific inquiry and the need to establish broader lines of argumentation that feed back into substantive theorizing.

Some of the socio-spatial relations that have been investigated – and will undoubtedly continue to yield fresh insights into the polymorphous geographies of contemporary state capitalism – include Sperber's (2022) complex relational geographies of social class undergirding state capitalism; political connections between state and business elites (Wood et al., 2023); geopolitics (Kinossian and Morgan, 2023; Ward et al., 2023); social relations of production and labor transformations (Alami and Dixon, 2023); relations of empire (Eagleton-Pierce, 2023; Silverwood and Berry, 2023; Whiteside, 2023); territorial relations at a range of scales (Su and Lim, 2023); networks of production (McGregor and Coe, 2023); corporate ownership (Babić, 2023; Babic et al., 2020; Haberly and Wójcik, 2017); and finance (Hall, 2023; Petry et al., 2023; Sokol, 2023). There is also considerable scope for further expanding the socio-spatial relations under consideration in state capitalism research, as some recent contributions have creatively shown (e.g. Medby on the relational construction of subjectivities and political identities of actors ‘performing’ state capitalism [in Alami et al., 2022b: 1014–1016]; or Liu and Dixon [2022] on the relational practices of the various professionals enacting state-capitalist investment). Future research could connect ecological political economy, the biophysical world and socio-metabolic processes to relational elements of state capitalism. This would open to a range of under-researched yet critical topics such as the place and limits of contemporary state capitalism in the energy transition (Alami et al., 2023; Babić and Dixon, 2022; Bridge and Gailing, 2020; Sweidan, 2021).

Not only an empirical exercise (that is, ‘thick descriptions’ of how relational patterns manifest) but relationality in state capitalism studies must also be analysed and theorized (Lejano and Kan, 2022). For Yeung (2005: 38), a robust theorization of the causes and power dynamics inherent to relationality offers a counterpart to more thematic treatments of relationality. State capitalism may well be in the midst of a ‘thematic turn,’ but theorization likely requires more sustained and conscious efforts to craft new ways of exposing and analysing a multiplicity of relationships, conceptualizing their nature and discerning their effects on the terraforming landscapes of state capitalism, including sustained reflection on methods and epistemology. In so doing, contributions to state capitalism would shift more overtly from the ‘what’ to the ‘how’ – from simply expanding the range of socio-spatial processes and relations investigated in state capitalism research, to reflections on how to precisely delineate research sites, units and objects of analysis when tracing relationality. The introductory commentary accompanying the third installment of the theme issue will make connections to methods of conjunctural analysis. More generally, contributions to state capitalism could begin to better specify why particular sets of socio-spatial relations are emphasized, how highly variegated socio-spatial relations relate to each other and to what extent agents’ socio-spatial ‘positionality’ shapes state-capitalist interactions and outcomes (Sheppard, 2002).

Embracing relationality, therefore, allows a significant redefinition of the *theoretical*, *empirical* and *spatial* parameters of the problematic of the new state capitalism. Geography moreover offers several leading spatial-relational frameworks for representing, mapping and theorizing relationality – from Gramscian geopolitical economy (Glassman, 2018) and régulation theory (Hillier et al., 2022) to network analysis (Coe and Yeung, 2019; Haberly and Wójcik, 2022), and actor–network and assemblage theory (Müller, 2015) – any/all of which have the potential to generate productive contributions. It is worth underlining that these spatial-relational frameworks hold widely divergent views on a series of key theoretical issues for state capitalism studies, such as the role and value of totality and abstraction. Some will see conceptual categories such as the state, capital, capitalism and uneven development as foundational elements to theorize state capitalism relationally, whereas others will likely find them unnecessarily totalizing. While such disagreements should make for constructive conversations in state capitalism studies, we note, however, that working with such abstractions may well be necessary, as argued in the first introductory essay to this theme issue (Whiteside et al., 2023). Yet this still leaves plenty of room for mobilizing, problematizing and developing relevant conceptual categories in a range of ways.

## Scaling the new state capitalism

Our second geographical provocation focuses on the notion of scale. Scale is not simply understood as a level of analysis (say, the local, urban, national and global). With Herod, we see scale as ‘the spatial resolution at which certain processes are understood to occur or particular entities are spatially constituted’ (Herod, 2020: 128). Scale is thus inseparable from time and space. Processes of capital accumulation and capitalist social relations are organized temporally, spatially and ‘scalarly’ (Herod, 2020). There is much debate as to the theoretical status and political role of scale in producing the geographies of capitalism, but one thing geographical political economists have shown is that geographical scales are socially constructed rather than ontologically pre-given or theoretically pre-ordained (Delaney and Leitner, 1997). More precisely, they are the product of material processes and political-economic struggles. For instance, different economic activities (e.g. labour markets, manufacturing capacities, industrial clusters, energy production and the circulation of capital in the built environment) tend to be scaled differently. Similarly, political authority – in the form of regulatory institutions, state authorities and circuits of policymaking – is constructed, negotiated, enforced and resisted at various scales of territorial organization. Importantly, these scalar arrangements are periodically rearranged, and the relations between them are reshuffled, often in the context of crises or as part of intense sociopolitical conflicts.

Relations between scales are indeed changing and fluid rather than fixed and immutable. Political-economic restructuring – such as the reorganization of governance hierarchies, the (de)globalization of economic flows, the re-regulation of financial activities, or the formation of new territorialized production complexes and markets – often involves (and may indeed be realized through) ‘rescaling’, that is the production of new scalar configurations. Such rescaling is achieved through, and results in, shifting constellations of power relations, the development of new organizational forms, and changing parameters of political action (Peck, 2002). State functions, spatial strategies and modalities of intervention undergo qualitative transformations through rescaling. Succinctly put, the scalar organization of capitalism is therefore something to be deciphered rather than taken for granted. The same is true of the new state capitalism.

This is important because overall, previous writings on state capitalism have arguably been insufficiently attentive to questions of scale. More precisely, much state capitalism research, largely due to its grounding in comparative institutionalism and the ‘varieties of capitalism’ approach, holds onto methodologically nationalist assumptions as to the scale of state capitalism. In line with these scholarly traditions and their common concern with national capitalisms *qua* distinct systems, state-capitalist models are assumed to be determined at the national scale, and primarily contained within national territories. Furthermore, these models are supposedly defined by a series of national institutional economic characteristics, such as the extent of state ownership in the national economy, the degree of state intervention in financial systems or in directing investment, the nature of domestic state-business relations, the extent of state protection of domestic markets from global competition, and others (see *inter alia*Fainshmidt et al., 2018; Kurlantzick, 2016; Nölke et al., 2019; Petry et al., 2023). Put simply, models of state capitalism differ from other varieties of capitalism in the ways in which they (nationally) mediate (global) market pressures. Thus, the key analytical units used to construct models of state capitalism have tended to be nationally indexed and constituted (nation-states, national economies, national-level or nationally anchored institutions and legislations, etc.).

Our point here is not that these writings exaggerate the importance of the nation-state as a geoeconomic and geopolitical scale, which remains of course hugely consequential in the scalar organization of global capitalism in general, and of state capitalism more specifically. Rather, the problem is that the national scale is taken as a self-evident empirical and theoretical focal point of state capitalism research. State capitalism research would do well to problematize this assumption so as to not miss equally significant processes unfolding at and between other scales (some of which we identify later on in this essay). Besides, if, as argued earlier, scales are indeed socially produced and fluid, then additional attention may be placed on those historical, geographic and institutional contingencies that are contributing to the ongoing reconstitution of the national scale, and the role this plays in changing forms and modalities of state intervention.

By re-envisioning contemporary state capitalism as a scaled phenomenon and process, and problematizing the national scale as a self-evident empirical and theoretical focal point of state capitalism, the scalar dynamics offered by geographical analyses shine a new light on state capitalism studies. Articles in this theme issue and elsewhere underline the significance of political-economic scales extending from the planetary divisions of labour (Alami and Dixon, 2023) to the scale of transcontinental empire (Silverwood and Berry, 2023; Whiteside, 2023), macro- and subnational regions (Peck, 2021; Su and Lim, 2023), municipalities (Paul and Cumbers, 2023) and residential and financial districts (Eagleton-Pierce, 2023; Hall, 2021; Ward et al., 2023). The picture that emerges from this work is not only one of present-day state capitalism as a spatially polymorphic and multi-scalar process (in contrast to state capitalism as monolithic bloc), these contributions equally indicate that *state-capitalist politics are fundamentally scalar politics* in at least two ways.

First, the state spatial strategies, institutional-regulatory and organizational forms and class-political projects associated with contemporary state capitalism can be seen as materializing at various scales of territorial organization and uneven development. Second, the social construction of multiple political-economic scales is itself an active moment in the constitution, evolution and contestation of state capitalism. Together these two aspects suggest that the new state capitalism involves ‘interscalar configurations’, where ‘scales are differentiated but intrinsically related, coevolving layers of territorial organization’ (Brenner, 2019: 129). In line with the case for relationality made earlier, adopting a scalar optic on state capitalism is not simply about considering the multiplicity of scales involved in the uneven (re)making of state capitalism and its socio-spatial power geometries, it is about thinking through their articulations. A relational and reflexive analysis of scale is, therefore, necessary here.

For instance, in their study of infrastructure projects financed by public-private partnerships, state enterprises and national development banks in Indonesia and the Philippines, Wijaya and Camba (2021) bring together a range of scale-differentiated social forces and processes: institutions of state capitalism simultaneously advance national and regional projects and the interests of domestically oriented business and political elites, help ‘fix’ transnational capital in space and deepen linkages with transnational class factions and international development agencies. As such, these infrastructure projects enable an interscalar re-articulation of state-capital relations. Ward et al. (2023) provide another insightful example of multi-scalar state capitalism in their article on Qatari sovereign fund investment in London where property markets and the built environment are shaped by: Qatar's attempt to project power transnationally (which is itself inseparable from domestic policy objectives and regional politics, notably fraught relations with its neighbor Saudi Arabia); the British state's post-imperial global ambition of retaining its centrality in global finance; and London government authorities seeking to address the city's housing crisis by creating investment vehicles to attract ‘patient’ state-capitalist investment. State capitalism, as foreign state-owned capital flows, does not neatly touch down in urban centres – urban space is materially and socially (re)ordered by multi-scalar state projects strategically coupled through investment.

By remaining attuned to scalar-relational approaches, geographical assessments more fully address the spatialities and temporalities underpinning the rise and significance of the new state capitalism by emphasizing the unstable transformations of entrenched scalar hierarchies, and the production of new tangled scalar arrangements. This is important insofar as the more visible role of the state as of late in the regulation of intra- and cross-border trade and capital flows (via state-led credit activism, capital controls, trade restrictions, investment screening mechanisms, technology bans and others) has often been misinterpreted, as mentioned earlier, as a sort of ‘return’ of national politics and re-assertion of nation-state sovereignty over globalization, financialization and the like (Ban and Bohle, 2021; Gerbaudo, 2021; Henow, 2022). Much like the way in which theorists of globalization during the 1990s tended to conflate the reconfiguration of the national scale of regulation with a withering of state power in the face of globalized market forces, there is a tendency to see the deployment of state-capitalist instruments and vehicles as a reversal of this trend. There is no doubt that we are witnessing a turbulent and crisis-ridden reshaping of nation, sovereignty and economic territory (more on this below), but this must not be understood as a zero-sum game between spatial scales of territorial organization, nor a unilateral process of enhanced state capacities to discipline markets. These apparently contradictory scalar tendencies call for analyses attentive to the complex and often ambivalent ways in which state strategies, discourses, institutions, modalities of intervention and targets, are being rescaled in a multiscalar fashion, and to what effect.

Looking ahead, we thus foresee and encourage further productive conversations between state capitalism and the literature on the political economy of scale, notably that on ‘state space’ and ‘scale rescaling’ (Brenner, 2019; Brenner et al., 2008), in order to develop a more nuanced understanding of both the destabilization of inherited scalar configurations and the production of new scalar fixes, as an expression of the dialectical relation between cycles of accumulation, institutional-regulatory restructuring and state (geo)political power projection. Such understanding will have to be able to concretely identify the specific political-economic mechanisms of rescaling associated with the new state capitalism.

Not without its challenges, a scalar-relational state capitalism calls for simultaneously connecting micro- and macro-geographies and moving between the particular and the whole/totality (Peck, 2016). Here, the meso-level and mid-range theorizing of economic geography may well be both a strength and a serious limitation. As a strength, mid-range and meso-level analysis illuminate locally specific, place-based processes and economic activities; however, limits arise when abstract-general forms of reasoning are eschewed (perhaps due to their association with structuralist theories). Moving between scales and levels of abstraction is precisely what is required here – not to reprioritize the general over the particular, or the macro over the micro, but instead to meet the call for linking contextualized inquiry with substantive theory-building, and negotiating the epistemological tension between the two, identified in the first Introduction to the theme issue (Whiteside et al., 2023). Enhanced considerations of state-capitalist scales and their relations to processes of uneven development may well require a renewed interest in dialectical-relational modes of thinking that mobilize various levels and modalities of abstraction (cf. Ollman, 2003), from theoretical elaboration to concrete exemplification and interrogation, although these will have to be infused and enriched with a scalar-relational sensitivity.

## Territorializing the new state capitalism

Our third and final geographical provocation focuses on the notion of territory. Territory, the theme issue demonstrates, is central to the problematic of new state capitalism in several respects. We already mentioned that state-capitalist transformations and strategies are not bound to the geographical borders of the nation-state given that they are scale-differentiated, stretch across space and cut across territorial economies. Territories are thus unequally affected by the operations of state capitalism. Here, we add that *the restructuring of territorial arrangements is itself a key vector of state capitalism*, and signature of novelty in ‘new’ state capitalism.

By contrast with conventional definitions which see territory as a bounded space (land, air space and waters) with fixed boundaries, under the juridiction or administeredd by a political authority, and which geographically contains a relatively coherent social formation, we follow geographical political economists in conceiving of territory as a distinctive mode of socio-spatial organization and a technology of power, which varies across geographical and historical contexts. As Elden (2010) puts it

Territory is a *historical* question: produced, mutable and fluid. It is *geographical*, not simply because it is one of the ways of ordering the world, but also because it is profoundly uneven in its development (Elden, 2010: 812; original emphasis).

We draw in particular on work that conceives of territory as both a (geo)political technology in the hands of states and as a (geo)economic technology of market-making in the hands of capital (Christophers, 2014). Territory is a geopolitical technology in the sense that it comprises techniques for establishing power relations of property, sovereignty and control over space, land, resources and environments (Elden, 2010). In its more explicitly geoeconomic dimension, the production of territory is part of the broader ‘spatial fix’ of capital accumulation (Harvey, 1982). Christophers (2014) writes that

Modern capitalism is constantly in the process of enacting territorial fixes: constituting, segmenting, differentiating and extracting value from actively territorialized markets at a range of geographical scales. These territorial fixes represent the market-oriented ‘face’ of the wider ‘scalar fixes’ … through which capital circulation has historically been constituted and reconstituted (Christophers, 2014: 755).

Territorialization, or the historical processes and social practices through which territory is constructed, enacted and enforced, often simultaneously involves both these geopolitical and geoeconomic dimensions, albeit at times in tension or outright contraction. With Lee et al. (2018), we indeed see territorialization as ‘bound up with the project of producing and reproducing capitalist (i.e. class) social relations, including the capitalist form of the state as a social relation’ (Lee et al., 2018: 416). Cases and sites of state capitalism research often constitute particularly visible, but perhaps no less antagonistic, entanglements of geopolitical and geoeconomic logics in the production of territory.

Consider the following examples. State-sponsored megaprojects, large-scale infrastructure and spatial planning strategies have been mobilized for territorial development and promotion, for integrating distant territories and facilitating the circulation of capital and for reconfiguring state sovereignty (Kinossian and Morgan, 2023; Su and Lim, 2023; Szabó and Jelinek, 2023). New forms of territorialized policy interventions have been deployed in order to compete in high-tech sectors, develop frontiers of resource extraction, attract foreign investment, catalyze structural change and achieve a range of state spatial objectives (Schindler et al., 2022; Wijaya and Camba, 2021; Zhang and Lan, 2023). Sovereign funds, state enterprises and other state-capital hybrids afford states the capacity to project geoeconomic power extra-territorially in transnational production, finance and digital networks (McGregor and Coe, 2023; Rolf and Schindler, 2023).

Focusing on these complex (re)territorializing logics, projects and strategies at a range of scales allows for the various forms of economic and geopolitical entanglement that constitute present-day state capitalism to be deciphered without lapsing into reified binaries. By moving away from immobile and bounded views of state power and territory (what Agnew described as the ‘territorial trap’ [1994]), more nuanced understandings of the reconfiguration of the state's role and/in uneven development come into focus. Take, for instance, the growing discourse of ‘decoupling’, ‘friendshoring’ and ‘nearshoring’, which have accelerated in the wake of the COVID-19 pandemic, the beginning of the war of aggression in Ukraine and the fracturing and repoliticization of trade relations between China and the United States. Commentators arguably have been quick to argue that those trends signal the end of globalization and the reorganization of economic activity around competing regional territorial blocs. There is undoubtedly some truth to that, in the sense that we are indeed witnessing clear attempts on the part of state and private actors to reorganize critical manufacturing and energy supply chains and control the diffusion of critical technologies. We also see more explicit (if not entirely transparent) attempts by states to leverage their economic positions to favour themselves over others, to craft a form of globalization more suited to their particular interests, and/or to corner third-party actors into their respective spheres of influence. Yet it is far from clear that most, if any, of the territorialization processes and practices which made globalization as we knew it in the 1990s and early 2000s – such as the organization of production complexes across borders, vast volumes of international trade and capital flows, deep global financial integration, the circulation of policies around extralocal policy networks, and the like – have come to an end. Rather than proclaiming the end of globalization, it might be wiser to speak of ‘complex overlayerings of territorializations and reterritorializations’ (Sparke, 2018: 487).

A similar mobilization of these conceptual categories of territory and territorialization could be productively deployed to extant and new subjects of state-capitalist investigation, such as the place of state-capitalist instruments in the (geo)political organization of the planetary circuits of capital, and their implications for dynamics of value extraction/appropriation/circulation and class struggles; geopolitical strategies and the structure of inter-capitalist competition, technological development and the distribution of financial risks and rewards; and geo-institutional variegation within and across world regions. Overall, this would contribute to our understandings of how territorial configurations spatially and politically mediate complex entanglements of geoeconomic and geopolitical logics, actors and processes.

Studying the dynamic production of territorial configurations also allows connecting agency with socio-spatial structure, insofar as they embody both the strategic dimension of state capitalism (the conscious design and implementation of state strategies and class-political projects to achieve particular ends) and structural imperatives (fragmented and precarious attempts at capturing spatial and scalar contradictions and containing crisis-prone developments). Hall’s (2021) analysis of efforts to make London an offshore hub for renminbi internationalization post-2008 is a case in point. Hall shows that aligning political and geoeconomic interests between various Chinese and British actors was achieved through the production of a particular ‘territorial fix’ in London's financial district. This fix embodied both structural and strategic imperatives. On the one hand, it was part of a broader state-led strategy to improve China's subordinate position in global financial and monetary relations, which is economically costly for Chinese firms and represents a serious political problem in a context of rapidly deteriorating US–China relations. On the other, it provided the British state with an opportunity to pivot towards China as a means to catalyze economic growth after the global financial crisis, and to cement the position of the City as a leading global financial centre.

Examples such as these suggest that matters of territory are not exclusive to interstate relations. Multiple actors, groups and social forces are involved in territorialization practices and processes, from policymakers to governmental and business factions in different sectors and policy domains, managers of state-owned enterprises and state investment funds, domestic and transnational corporate elite communities, financial professionals, organized labour organizations, civil society groups and so on.

Finally, a keen attention to articulations of space and territory can help refining our understanding of the multiple temporal geographies of contemporary state capitalism*.* We have already touched upon thorny questions of periodization, temporalities and temporal unevenness in previous writings (see Alami and Dixon, 2023; Whiteside et al., 2023). Here we add that further studying dynamics of (re)territorialization over time can yield new insights into the evolving character of state capitalism, including patterns of continuity, rupture and transformation. In this vein, Silverwood and Berry (2023) make clear that British state capitalism is a transnational project centred variably on empire, Europe and the global market; a sentiment shared by Eagleton-Pierce (2023) with reference to the City of London Corporation. Contributions such as these demonstrate that scrutinizing the territorial transformations of state capitalism over longer historical arc helps us understand that a capacity to support shifting modes of capital accumulation and reproduction of class power relies on a combination of path-dependencies (in institutional, political, socio-spatial and cultural terms) and innovations (including the reinvention of spatialized forms of state intervention, institutional-regulatory experimentation and the like). Another example is the work of Lee (in Alami et al., 2022b: 1011–1014), which shows that being attentive to the production of territory allows one to rethink some of the temporalities of the new state capitalism: while East Asian state capitalism is often associated with the temporalities of ‘catch-up’ development (i.e. development ‘laggards’ attempting to close the productivity gap with more advanced capitalist economies), zoning, territorialized industrial policies and other flexible territorial arrangements in South Korea are actually geared towards making specific regions world bleaders in the digital sectors associated with the ‘Fourth Industrial Revolution’.

In conclusion, though not without its challenges, the recent turn towards spatiotemporal relationality and uneven development in new state capitalism studies has opened a rich field of possibility for comparison and critical enquiry. The contributions to this three-part theme issue illustrate numerous ways in which these debates can be taken forward. In the interest of consolidating some of these emerging currents in state capitalism studies, we have attempted in this essay to show what it would mean – in theoretical, epistemological and methodological terms – more fully to integrate some key tools of critical socio-spatial analysis, namely relationality, scale and territory, into the conceptual grammar of state capitalism studies. Mobilizing these tools, we maintain, not only allows for the deepening of spatial reflexivity in state capitalism studies (linked to more or less sympathetic critiques of previous approaches), it also reconfigures the theoretical, empirical, spatiotemporal coordinates of the problematic of the ‘new’ state capitalism itself. Our final introductory commentary (to part three of the theme issue) will explore how methods of conjunctural analysis can be mobilized in a new generation of state capitalism studies.

## References

[bibr1-0308518X231156913] Agnew J (1994) The territorial trap: The geographical assumptions of international relations theory. Review of International Political Economy 1(1): 53–80.

[bibr2-0308518X231156913] Alami I (2022) The spiral of state capitalism: Labour transformations or the ‘whip of external necessity’? Global Political Economyearly view.

[bibr3-0308518X231156913] Alami I Dixon AD (2023) Uneven and combined state capitalism. EPA: Economy and Space 55(1): 72–99.

[bibr4-0308518X231156913] Alami I Babić M Dixon AD , et al. (2022a) Special issue introduction: What is the new state capitalism? Contemporary Politics 28(3): 245–263.

[bibr5-0308518X231156913] Alami I Dixon AD Gonzalez-Vicente R , et al. (2022b) Geopolitics and the ‘new’ state capitalism. Geopolitics 27(3): 995–1023.

[bibr6-0308518X231156913] Alami I Copley J Moraitis A (2023) The ‘wicked trinity’ of late capitalism: Governing in an era of stagnation, surplus humanity, and environmental breakdown. Geoforum; Journal of Physical, Human, and Regional Geosciencesearly view.

[bibr7-0308518X231156913] Anievas A Nisancioglu K (2015) How the West Came to Rule: The Geopolitical Origins of Capitalism. London: Pluto Press.

[bibr8-0308518X231156913] Babić M (2023) The Rise of State Capital: Transforming Markets and International Politics. Newcastle Upon Tyne: Agenda Publishing.

[bibr9-0308518X231156913] Babić M Dixon AD (2022) Decarbonising states as owners. New Political Economyearly view.

[bibr10-0308518X231156913] Babic M Garcia-Bernardo J Heemskerk EM (2020) The rise of transnational state capital: State-led foreign investment in the 21st century. Review of International Political Economy 27(3): 433–475.

[bibr11-0308518X231156913] Baltz MJ (2022) What lies beneath the ‘tariff man’? The Trump administration’s response to China’s ‘state capitalism’. Contemporary Politics 28(3): 328–346.

[bibr12-0308518X231156913] Ban C Bohle D (2021) Definancialization, financial repression and policy continuity in East-Central Europe. Review of International Political Economy 28(4): 874–897.

[bibr13-0308518X231156913] Brenner N (2019) New Urban Spaces: Urban Theory and the Scale Question. Oxford: Oxford University Press.

[bibr14-0308518X231156913] Brenner N Jessop B Jones M , et al. (eds) (2008) State/Space: A Reader. Oxford: John Wiley & Sons.

[bibr15-0308518X231156913] Bridge G Gailing L (2020) New energy spaces: Towards a geographical political economy of energy transition. EPA: Economy and Space 52(6): 1037–1050.

[bibr16-0308518X231156913] Christophers B (2014) The territorial fix: Price, power and profit in the geographies of markets. Progress in Human Geography 38(6): 754–770.

[bibr17-0308518X231156913] Coe NM Yeung HWC (2019) Global production networks: Mapping recent conceptual developments. Journal of Economic Geography 19(4): 775–801.

[bibr18-0308518X231156913] Delaney D Leitner H (1997) The political construction of scale. Political Geography 16(2): 93–97.

[bibr19-0308518X231156913] Eagleton-Pierce M (2023) Uncovering the City of London Corporation: Territory and temporalities in the new state capitalism. EPA: Economy and Space 55(1): 184–200.

[bibr20-0308518X231156913] Elden S (2010) Land, terrain, territory. Progress in Human Geography 34(6): 799–817.

[bibr21-0308518X231156913] Fainshmidt S Judge WQ Aguilera RV , et al. (2018) Varieties of institutional systems: A contextual taxonomy of understudied countries. Journal of World Business 53(3): 307–322.

[bibr22-0308518X231156913] Gerbaudo P (2021) The Great Recoil: Politics After Populism and Pandemic. London: Verso.

[bibr23-0308518X231156913] Germann J (2022) Global rivalries, corporate interests and Germany’s ‘National Industrial Strategy 2030’. Review of International Political Economyearly view.

[bibr24-0308518X231156913] Glassman J (2018) Geopolitical economies of development and democratization in East Asia: Themes, concepts, and geographies. EPA: Economy and Space 50(2): 407–415.

[bibr25-0308518X231156913] Haberly D Wójcik D (2017) Earth incorporated: Centralization and variegation in the global company network. Economic Geography 93(3): 241–266.

[bibr26-0308518X231156913] Haberly D Wójcik D (2022) Sticky Power: Global Financial Networks in the World Economy. Oxford: Oxford University Press.

[bibr27-0308518X231156913] Hall S (2021) Respatialising Finance: Power, Politics and Offshore Renminbi Market Making in London. Oxford: John Wiley & Sons.

[bibr28-0308518X231156913] Hall S (2023) Locating state capitalism: Financial centres and the internationalisation of Chinese banks in London. EPA: Economy and Spaceearly view.

[bibr29-0308518X231156913] Harvey D (1982) Limits to Capital. London: Verso.

[bibr30-0308518X231156913] Harvey D (2005) A Brief History of Neoliberalism. Oxford: Oxford University Press.

[bibr31-0308518X231156913] Henow A (2022) Helpless victim of financialisation? Financial liberalisation, crisis and taking back control in South Korea. Cambridge Journal of Economics 46(5): 1161–1182.

[bibr131-0308518X231156913] Herod A (2020) Time, space, geographical scale and political economy. In: Dunn B (ed) *A Research Agenda for Critical Political Economy.* Cheltenham, UK: Edward Elgar Publishing. pp. 121–133.

[bibr32-0308518X231156913] Hillier B Phillips R Peck J (eds) (2022) Regulation Theory, Space, and Uneven Development: Conversations and Challenges. Vancouver: 1984press.

[bibr33-0308518X231156913] Jensen F (2023) State capitalism and Spanish port development along the Maritime Silk Road. EPA: Economy and Space 55(3): 636–654.

[bibr34-0308518X231156913] Jones M (2009) Phase space: Geography, relational thinking, and beyond. Progress in Human Geography 33(4): 487–506.

[bibr35-0308518X231156913] Kinossian N Morgan K (2023) Authoritarian state capitalism: Spatial planning and the megaproject in Russia. EPA: Economy and Space 55(3): 655–672.

[bibr36-0308518X231156913] Kurlantzick J (2016) State Capitalism: How the Return of Statism is Transforming the World. Oxford: Oxford University Press.

[bibr37-0308518X231156913] Lee SO Wainwright J Glassman J (2018) Geopolitical economy and the production of territory: The case of US–China geopolitical-economic competition in Asia. Environment and Planning A: Economy and Space 50(2): 416–436.

[bibr38-0308518X231156913] Lejano RP Kan WS (2022) Relationality: The Inner Life of Public Policy. Cambridge: Cambridge University Press.

[bibr39-0308518X231156913] Liu IT Dixon AD (2022) What does the state do in China’s state-led infrastructure financialisation? Journal of Economic Geography 22(5): 963–988.

[bibr40-0308518X231156913] McGregor N Coe NM (2023) Hybrid governance and extraterritoriality: Understanding Singapore’s state capitalism in the context of oil global production networks. EPA: Economy and Space 55(3): 716–741.

[bibr41-0308518X231156913] Massey D (1984) Spatial Divisions of Labour: Social Structures and the Geography of Production. Basingstoke: Macmillan.

[bibr42-0308518X231156913] Müller M (2015) Assemblages and actor-networks: Rethinking socio-material power, politics and space. Geography Compass 9(1): 27–41.

[bibr43-0308518X231156913] Murphy JT (2018) The relational turn in economic geography. In: Cook G Johns J McDonald F Beaverstock J Pandit N (eds) The Routledge Companion to the Geography of International Business. London: Routledge, pp. 161–173.

[bibr44-0308518X231156913] Nölke A Ten Brink T May C , et al. (2019) State-permeated Capitalism in Large Emerging Economies. London: Routledge.

[bibr45-0308518X231156913] Ollman B (2003) Dance of the Dialectic: Steps in Marx’s Method. Urbana: University of Illinois Press.

[bibr46-0308518X231156913] Paul FC Cumbers A (2023) The return of the local state? Failing neoliberalism, remunicipalisation, and the role of the state in advanced capitalism. EPA: Economy and Space 55(1): 165–183.

[bibr47-0308518X231156913] Peck J (2002) Political economies of scale: Fast policy, interscalar relations, and neoliberal workfare. Economic Geography 78(3): 331–360.

[bibr48-0308518X231156913] Peck J (2016) Macroeconomic geographies. Area Development and Policy 1(3): 305–322.

[bibr49-0308518X231156913] Peck J (2021) On capitalism’s cusp. Area Development and Policy 6(1): 1–30.

[bibr50-0308518X231156913] Peck J (2023) Variegated Economies. Oxford: Oxford University Press.

[bibr51-0308518X231156913] Petry J Koddenbrock K Nolke A (2023) State capitalism and capital markets: Comparing securities exchanges in emerging markets. EPA: Economy and Space 55(1): 146–164.

[bibr52-0308518X231156913] Plant R (2010) The Neo-liberal State. Oxford: Oxford University Press.

[bibr53-0308518X231156913] Ricz J (2021) The anatomy of the newly emerging illiberal model of state capitalism: A developmental dead end? International Journal of Public Administration 44(14): 1253–1263.

[bibr54-0308518X231156913] Rogers S (2022) Illiberal capitalist development: Chinese state-owned capital investment in Serbia. Contemporary Politics 8(3): 347–364.

[bibr55-0308518X231156913] Rolf S Schindler S (2023) The US-China rivalry and the emergence of state platform capitalism. EPA: Economy and Spaceearly view.

[bibr56-0308518X231156913] Schindler S Alami I Jepson N (2022) Goodbye Washington confusion, hello Wall Street consensus: Contemporary state capitalism and the spatialisation of industrial strategy. New Political Economyearly view.

[bibr57-0308518X231156913] Sheppard E (2002) The spaces and times of globalization: Place, scale, networks, and positionality. Economic Geography 78(3): 307–330.

[bibr58-0308518X231156913] Sheppard E (2011) Geographical political economy. Journal of Economic Geography 11(2): 319–331.

[bibr59-0308518X231156913] Silverwood J Berry C (2023) The distinctiveness of state capitalism in Britain: Market-making, industrial policy and economic space. EPA: Economy and Space 55(1): 122–142.

[bibr60-0308518X231156913] Sokol M (2023) Financialisation, central banks and the ‘new’ state capitalism: The case of the US Federal Reserve, the European Central Bank and the Bank of England. EPA: Economy and Spaceearly view.

[bibr61-0308518X231156913] Sparke M (2018) Globalizing capitalism and the dialectics of geopolitics and geoeconomics. EPA: Economy and Space 50(2): 484–489.

[bibr62-0308518X231156913] Sperber N (2022) Servants of the state or masters of capital? Thinking through the class implications of state-owned capital. Contemporary Politics 28(3): 264–284.

[bibr163-0308518X231156913] Su X and Lim KF (2023) Capital accumulation, territoriality, and the reproduction of state sovereignty in China: Is this “new” state capitalism? *EPA: Economy and Space*.

[bibr63-0308518X231156913] Sweidan OD (2021) State capitalism and energy democracy. Geoforum; Journal of Physical, Human, and Regional Geosciences 125: 181–184.

[bibr64-0308518X231156913] Swyngedouw E (2000) The Marxian alternative: Historical-geographical materialism and the political economy of capitalism. In: Sheppard E Barnes TJ (eds) A Companion to Economic Geography. Oxford: Wiley, pp. 41–59.

[bibr65-0308518X231156913] Szabó L Jelinek C (2023) State, capitalism and infrastructure-led development: A multi-scalar analysis of the Belgrade-Budapest railway construction. EPA: Economy and Spaceearly view.

[bibr66-0308518X231156913] Ward C Brill F Raco M (2023) State capitalism, capitalist statism: Sovereign wealth funds and the geopolitics of London’s real estate market. EPA: Economy and Space 55(3): 742–759.

[bibr67-0308518X231156913] Whiteside H (2023) Company colonies and historical layering: Understanding the Virginia, Somers Isles, and Hudson’s Bay Companies. Review of International Political Economyearly view.

[bibr68-0308518X231156913] Whiteside H Alami I Dixon AD , et al. (2023) ‘Making space for the new state capitalism’, part I: Working with a troublesome category. EPA: Economy and Space 55(1): 63–71.

[bibr69-0308518X231156913] Wijaya T Camba A (2021) The politics of public-private partnerships: State-capital relations and spatial fixes in Indonesia and the Philippines. Territory, Politics, Governanceearly view.

[bibr70-0308518X231156913] Wood GT Onali E Grosman A , et al. (2023) A very British state capitalism: Variegation, political connections and bailouts during the COVID-19 crisis. EPA: Economy and Space 55(3): 673–696. 10.1177/0308518X211072545 PMC10172842 37192929

[bibr71-0308518X231156913] Yeung HWC (2005) Rethinking relational economic geography. Transactions of the Institute of British Geographers 30(1): 37–51.

[bibr72-0308518X231156913] Zhang L Lan T (2023) The new whole state system: Reinventing the Chinese state to promote innovation. EPA: Economy and Space 55(1): 201–221.

